# Electrospun nanofiber membrane diameter prediction using a combined response surface methodology and machine learning approach

**DOI:** 10.1038/s41598-023-36431-7

**Published:** 2023-06-15

**Authors:** Md. Nahid Pervez, Wan Sieng Yeo, Mst. Monira Rahman Mishu, Md. Eman Talukder, Hridoy Roy, Md. Shahinoor Islam, Yaping Zhao, Yingjie Cai, George K. Stylios, Vincenzo Naddeo

**Affiliations:** 1 Hubei Provincial Engineering Laboratory for Clean Production and High Value Utilization of Bio-Based Textile Materials, Wuhan Textile University, Wuhan, 430200 China; 2 Sanitary Environmental Engineering Division (SEED), Department of Civil Engineering, University of Salerno, 84084 Fisciano, Italy; 3 Department of Chemical and Energy Engineering, Faculty of Engineering and Science, Curtin University Malaysia, CDT 250, 98009 Miri, Sarawak Malaysia; 4 Faculty of Nutrition and Food Science, Patuakhali Science and Technology University, Patuakhali, 8602 Bangladesh; 5 Department of Chemical Engineering, Bangladesh University of Engineering and Technology, Dhaka, 1000 Bangladesh; 6 Shanghai Engineering Research Center of Biotransformation of Organic Solid Waste, School of Ecological and Environmental Sciences, East China Normal University, and Institute of Eco-Chongming, Shanghai, 200241 China; 7 Research Institute for Flexible Materials, School of Textiles and Design, Heriot-Watt University, Galashiels, TD1 3HF UK

**Keywords:** Materials science, Nanoscale materials

## Abstract

Despite the widespread interest in electrospinning technology, very few simulation studies have been conducted. Thus, the current research produced a system for providing a sustainable and effective electrospinning process by combining the design of experiments with machine learning prediction models. Specifically, in order to estimate the diameter of the electrospun nanofiber membrane, we developed a locally weighted kernel partial least squares regression (LW-KPLSR) model based on a response surface methodology (RSM). The accuracy of the model's predictions was evaluated based on its root mean square error (*RMSE*), its mean absolute error (*MAE*), and its coefficient of determination (*R*^2^). In addition to principal component regression (PCR), locally weighted partial least squares regression (LW-PLSR), partial least square regression (PLSR), and least square support vector regression model (LSSVR), some of the other types of regression models used to verify and compare the results were fuzzy modelling and least square support vector regression model (LSSVR). According to the results of our research, the LW-KPLSR model performed far better than other competing models when attempting to forecast the membrane's diameter. This is made clear by the much lower *RMSE* and *MAE* values of the LW-KPLSR model. In addition, it offered the highest R^2^ values that could be achieved, reaching 0.9989.

## Introduction

Nanofibers are fibrous materials characterized by their diameter, typically within the 1 to 100 nm range. The nanofibers exhibit a significant surface area, elevated aspect ratio, exceptional surface characteristics, quantum confinement phenomena, and rapid biomolecule absorption capabilities, resulting in applications including environmental engineering, biomedical engineering, tissue engineering, mechanical engineering, etc.^1,2^. Electrospinning is a simple approach that may be used to manufacture polymeric nanofibers from a broad range of polymers in the presence of a strong electrostatic field^3–6^. A pump, a syringe equipped with a nozzle, a power source for the electric field, and either a reference electrode or an item that is grounded make up the fundamental components of the electrospinning device. The nozzle of the syringe and the counter electrode, where the solution that will be electrospun is maintained, work together to generate a strong electrical field. As the charged jet accelerates toward the counter electrode, the solvent in the solution evaporates, forming solid continuous nanofibers on the grounded target. As a result of the difference in potential between the ejector and the grounded target, the form of the solution droplet that is blasted from the nozzle is distorted into that of a cone^7,8^. The diameter of the nanofibers plays a vital role in determining their functionality, such as adsorption, filtration, catalytic degradation and so on^9,10^. Importantly, several factors, such as the concentration of the polymer solution, the surface tension, the viscosity, the conductivity, the flow rate, the voltage, the distance between the needle and the collector, the needle size, and the relative humidity, influence the diameter variation. As a result, the following parameters must be carefully adjusted to produce ideal nanofibers membrane diameters with their functional properties^11,12^.

The traditional one-factor-at-a-time method (OFAT) optimization method is expensive and time-consuming, and it may not always lead to the identification of optimal values for all variables. Design of Experiment (DOE) has shown to be an excellent method for planning and enhancing experiments. It's a reliable statistical method for maximizing the output of experiments using known inputs. It is necessary to optimize the information acquired while limiting the experimental n-number of runs^13–16^. In order to build an approximate model that describes the connection between a response and a collection of predictor variables, the response surface methodology (RSM) is a straightforward and effective optimization approach. The most popular RSM optimization approach for maximizing output while minimizing input is the Box–Behnken design (BBD). This method is based on factorial design and employs a box-shaped unfinished block with a wireframe composing the box's inside. With the BBD, users may fit a second-order polynomial model to the data by utilizing the mathematical connection between the variables and the responses^17^. Central composite design (CCD) is another popular RSM method for analyzing the impact of variable inputs on output quality. The CCD quadratic model plays an important role in the RSM framework because it displays many curved functions^18^. Numerous research efforts have concentrated on BBD and CCD-based RSM strategies for regulating the electrospun nanofiber's diameter^19–21^. Due to the nonlinear input–output correlations in process data, experimental researchers often find RSM a challenging tool^22^.

As a result, machine learning (ML) models may emerge as a viable alternative to the RSM. It is used in many fields because it boosts the efficiency of statistical optimization techniques, reducing the time and money needed for simulations^23,24^. Partial least squares (PLS) modelling is widely used since it is easy to understand and can easily extract characteristics from data^25–27^. PLS has had widespread use, although it is not without limitations. PLS is limited in its ability to gather data features by the inherent linearity of the method, which becomes problematic when the relationship between the data is not linear. Although, in complex chemical processes, nonlinearity between process variables is typical. As a consequence, several nonlinear upgrades have been proposed to include nonlinear properties inside the framework of linear PLS^28^. An adaptive version of locally weighted partial least squares (LW-PLS) was introduced to account for the fluctuating degrees of nonlinearity between input and output variables in the context of a specific query. The collinearity problem between process variables may be solved by projecting process data onto low-dimensional latent variables while maintaining the full information stored within input and output data. However, most local regression methods are not flexible enough to account for changes in nonlinearity. This makes them somewhat unreliable. Furthermore, conventional local regression methods continue to use the original process variables as input features, and nonlinearity in regression models is predicated only on the unique treatment of samples (i.e., local weighting). As a result, the accuracy of forecasts made using conventional methods could not live up to expectations, especially for highly nonlinear industrial processes^29,30^.

A novel sparse nonlinear feature-based regression method called locally weighted kernel partial least squares (LW-KPLSR) is proposed in a series of sentences as a reasonable solution to the abovementioned issues. The sparse kernel feature characterisation factors are used in the proposed LW-KPLSR method to establish the relative importance of each training sample, in contrast to the classic LW-PLS^31,32^. In a recent study, Yeo et al.^33^ demonstrated implementing the LW-KPLSR model for adaptive soft sensors. It was found that there are distinctions between LW-PLS and LW-KPLSR and correspond to expected differences in efficiency and computation requirements. The results show that the prediction performance of LW-KPLSR is higher than that of LW-PLS. Furthermore, Zhang et al.^31^ presented a special LW-KPLS based on sparse nonlinear features subjected to virtual exhibits through nonlinear time-varying processes. The suggested LW-KPLSR improves upon the locally weighted (LW) technique's capacity to cope with extreme nonlinearity by including the non-linear feature into the locally weighted framework. LW-KPLSR models, which account for non-linear dependencies in both the training samples and the query, are constructed using the ideas of sparse Kernel feature characterization. Incorporating nonlinear features into the locally weighted regression framework, the suggested LW-KPLSR is more adapted to highly nonlinear processes and more adept at handling time-varying characteristics than the conventional LW-PLS. Following this, several scientists have suggested different empirical relationships to account for all the variables that affect electrospinning^34^. In order to construct certain empirical scaling laws of parameters and diameter, Wang et al.^35,36^ and Yousefi et al.^37^ employed experiments as their primary data source. On the other hand, the applicability of the empirical model is severely limited since there is not enough systematization and characterization^38^. If a flawless mathematical and physical model for electrospinning simulation can be developed, it will be possible to make precise predictions about the form and characteristics of nanofibers. This can potentially improve the quantity of work researchers can accomplish and open the way to a larger variety of applications for electrospinning technology. However, as of yet, no work has concentrated on the prediction of the diameter of electrospun nanofibers by using the LW-KPLSR model.

In light of this, this study aims to combine the RSM optimization model with the machine learning model to predict the diameter of electrospun nanofiber membranes using three case studies, a relatively novel technique in scientific literature. Following that, we compute the model's root mean squared error (*RMSE*), absolute mean error (*MAE*), and coefficient of determination to establish the level of accuracy it has (*R*^2^). We also look at the outcomes of the fuzzy model, principal component regression (PCR), and the least square support vector regression model (LSSVR) to better understand the different models' capacities for making an accurate prediction.

## Experimental

### Materials and methods

The RSM design optimization approach, the electrospinning process, and the nanofiber membrane's diameter measurement are all topics covered in this part. The subsequent step describes the machine learning models, establishing the regression models' parameters and assessing the prediction performance's correctness.

### RSM-aided electrospinning

#### Case study 1: Poly(vinyl alcohol)/chitosan crosslinked electrospun nanofiber membrane

**Figure 1:**
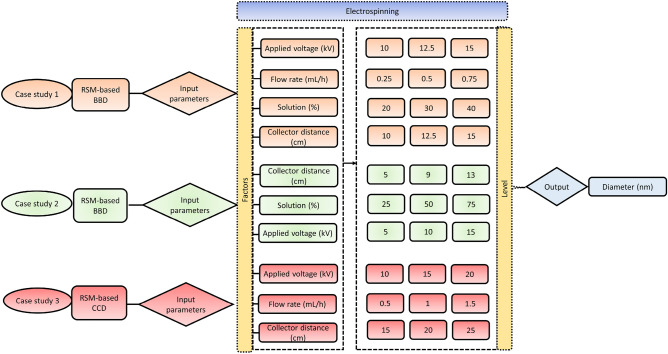
A layout of the RSM-aided electrospinning processing parameters optimization and response strategy.

The experimental work of Viana et al.^39^ provided the data used for training and evaluating the models in Case Study 1. This study used a BBD (four-factor and three-level) to find the optimum conditions under which the mean diameter of the nanofibers was reduced to its minimum (Fig. 1). A 5 mL disposable syringe fitted with a 0.55 mm internal diameter disposable needle (25G) and a pump capable of regulating solution injection flow was filled with the prepared PVA: CS solutions. The metal needle was linked to a high-voltage source producing 60 kV at its tip, and a metal plate was connected to the ground wire and held at a certain distance from the needle. A temperature of 25˚C and relative humidity of 50% -60% were ideal for electrospinning. Additionally, the samples were dried in a vacuum desiccator with 15 mL of 25% aqueous glutaraldehyde solution on a Petri dish for 24 h to fabricate crosslinked PVA: CS nanofibrous membranes.

#### Case study 2: Chitosan-based electrospun nanofiber membrane

In case study 2, data used for model training and testing were collected from the experimental work of Thirugnanasambandham et al.^40^. The Ag/CS colloid and PEO were dissolved in a 3 wt.% acetic acid solution in water, and the mass ratio of CS to PEO was set at 1:1, yielding a solution with a polymer concentration of 10 wt%. A control solution of ordinary CS/PEO with the same concentration was also compared. A syringe pump was used to regulate the delivery of the polymer solution at a constant rate of 0.1 mL/h while the voltage and collector distance were changed. A metal capillary (ID = 0.5 mm) within a syringe was used to push the prepared solution out with the help of a DC power source. The nanofibrous were stored in a desiccator for 5 h after cross-linking with a 25% (w/v) glutaraldehyde aqueous solution at 35 °C. Figure 1 depicts the scheme of an investigation into the electrospinning process using RSM in conjunction with a BBD.

#### Case study 3: Chitosan-collagen electrospun nanofiber membrane

In case study 3, data for model training and testing were obtained through the experimental work of Amiri et al.^41^. Chitosan and collagen were dissolved in acetic acid at a 90% (v/v) concentration to make a chitosan-collagen solution. The final product had a total of 5% collagen and 2.5% chitosan. To promote electrospinning, a PEO solution (2.5 wt%) was added to a chitosan-collagen solution (10:90 volume ratio). In order to electrospun the chitosan-collagen solution, a 5 ml syringe was placed horizontally on a revolving drum coated in aluminium foil, and an 18 G stainless steel needle was used to provide electric current. As can be seen in Fig. 1, the electrospinning process followed an RSM-coupled CCD approach.

As a whole, Image J, an open-access software, was used to evaluate and quantify nanofibre diameter. The United States National Institutes of Health (NIH) hosts the downloadable version of this program online (https://imagej.nih.gov/ij/download.html).

### Machine learning approach

The regression models used were LSSVR, PCR, PLSR, LW-PLSR, LW-KPLSR, and Fuzzy method, in which the diameter of the electrospun nanofiber membrane was referred to as the output variable for each case study. In the first case study, a total of 27 electrospinning data sets were employed as the input variables for the regression models. These data sets included the applied voltage, flow rate, chitosan solution concentration, and tip-to-needle distance (Table S1). For the second case study, there were a total of 17 different sets of electrospinning data that were utilized as input variables for the regression models. These sets included information on collector distance, polymer solution concentration, and applied voltage (Table S2). In the third case study, 20 different sets of electrospinning data were used as the input variables for the regression models. These data sets included the applied voltage, flow rate, and needle-to-collector distance (Table S3).

**Figure 2:**
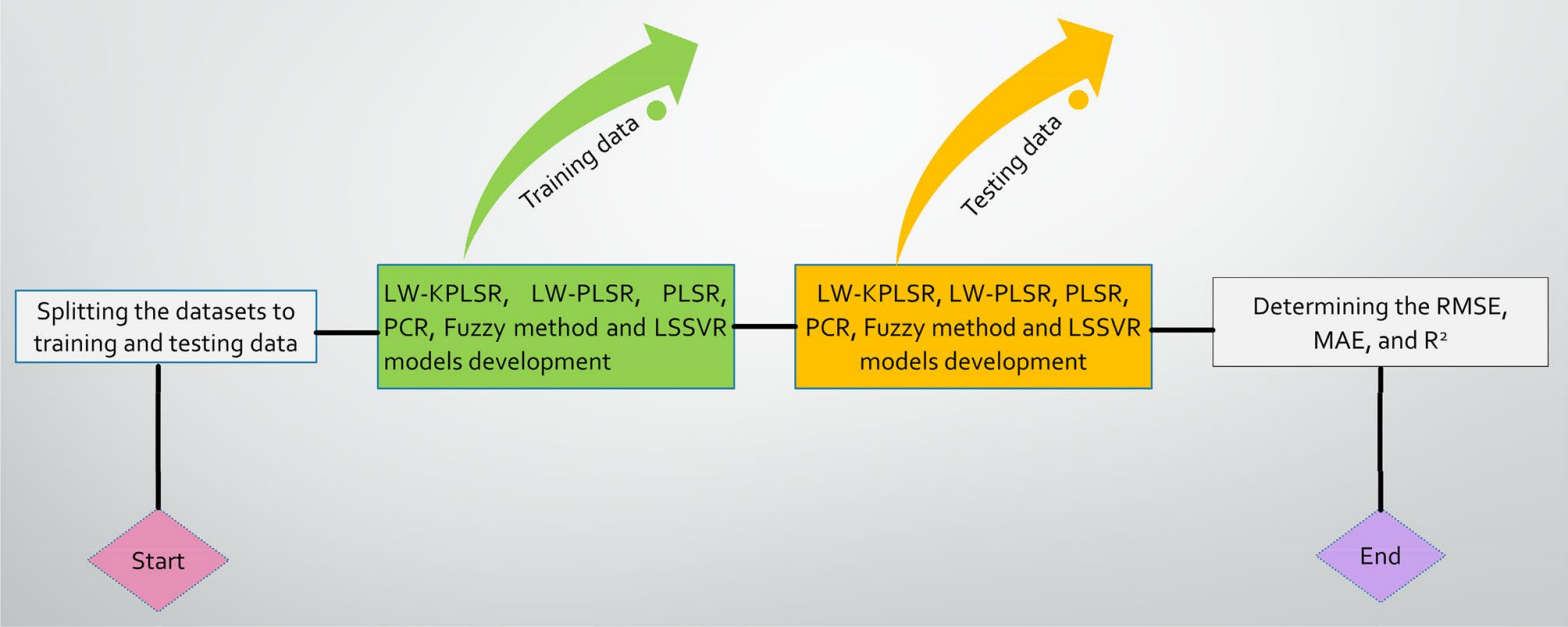
A framework of LW-KPLSR, LW-PLSR, PLSR, PCR, Fuzzy method and LSSVR models development.

Once the data was imported into MATLAB for each case study, it was then divided between the training set and the testing set in an 80/20 ratio (Tables S4–S6). The general layout of the six distinct regression models (LW-KPLSR, LW-PLSR, PLSR, PCR, Fuzzy approach, and LSSVR) is shown to predict nanofiber diameter values in Fig. 2. These models are utilized to estimate nanofiber diameter values. The LW-KPLSR, LW-PLSR, PLSR, PCR, Fuzzy approach, and LSSVR models were used to assess the progress of the models using the same models that were used during training and testing, as shown in Fig. 2. Table 1 provides an overview of the several possible parameter settings that may be used with the LW-KPLSR, LW-PLSR, PLSR, PCR, Fuzzy approach, and LSSVR models. The total number of datasets, the number of datasets used for training, the number of datasets used for testing, and the number of latent variables are denoted by N_T_, N_1_, N_2_ and LV correspondingly. During this time, the tuning parameters used in the LSSVR model are denoted by the symbols γ, λ, and p. Additionally, b is the kernel parameter utilized in the kernel functions, and all of these parameters were adjusted.

**Table 1:** Values used for LW-KPLSR, LW-PLSR, PLSR, PCR, Fuzzy method and LSSVR models.

Parameters	${\mathrm{N}}_{\mathrm{T}}$	${\mathrm{N}}_{1}$	${\mathrm{N}}_{2}$	LV	γ	λ	*p*	*b*
Values (Case study 1)	27	18	9	1	128	1	4.8828 × 10^–4^	0.01
Values (Case study 2)	17	11	6	1	0.0313	9.7656 × 10^–4^	2	0.01
Values (Case study 3)	20	13	7	1	8192	9.7656 × 10^–4^	3.0518 × 10^–5^	0.01

### Analysis of prediction behaviour

*RMSE* is a scale-dependent defect measure that assesses whether a prediction model is successful. This statistic was used to ascertain whether the data splitting ratio was satisfactory since it enabled comparisons across different setups for a single variable. The accuracy of a forecast is measured by a statistic called *RMSE*, where a smaller number indicates better accuracy. *RMSE* is found by adding up all of the squared discrepancies between observed and predicted values^42^. It might also be seen as a reflection of the discrepancies between predicted and observed values. A smaller *RMSE* indicates a higher degree of accuracy and predictive power when this is considered. Equation (1)^43^ displays the *RMSE* formula.

1 $$ RMSE = \sqrt {\frac{{\sum\nolimits_{i} {\left( {Y_{i} - \hat{Y}_{i} } \right)}^{2} }}{n}} $$

The $Y_{i}$ represents the actual output, $\hat{Y}_{i}$ stands for the predicted output, and n represents the total number of samples.

As demonstrated in Eq. (2), the *MAE* is a statistic for assessing a collection of predictions that disregards both the directionality and severity of mistakes. For each observation in the test data set, this value represents the weighted mean of the absolute discrepancies between the predicted and actual values.

2 $$MAE=\frac{1}{n}{\sum }_{j=1}^{n}|{Y}_{i}-{\widehat{Y}}_{i}|$$

where $\sum {}$ represents the summation.

*R*^*2*^ is a measurement that determines how effectively a regression model accounts for the variance in the target variable present in a particular dataset^42^. *R*^2^ is a statistical metric used to determine the "goodness of fit" between the observed values and predicted in a regression model. Its value might fall anywhere between 0 and 1^44^. If it is close to one, it indicates that the chosen inputs should yield the intended output; if it is farther away, it indicates that the fit may need some adjustment. Finding the *R*^2^ involves comparing the sum of squared errors and the total of squared deviations from the mean of the variable under investigation. A statistic known as *R*^2^ is used to quantify the degree to which data that has been seen matches data that has been anticipated. Equation (3)^45^ provides the whole explanation of the formula.

3 $$ R^{2} = 1 - \frac{{\sum\nolimits_{i} {\left( {Y_{i} - \hat{Y}_{i} } \right)}^{2} }}{{\sum\nolimits_{i} {\left( {Y_{i} - \overline{Y}} \right)^{2} } }} $$

To be more explicit, Eq. (4)^46^ offers a mathematical explanation of the prediction error (*PE*) that is used in the process. To offer quantitative proof of the predictive capacities of diameter values, we analyze the error of approximation, which is expressed by *E*_*a*_, and calculate *E*_*a*_ using Eq. (5)^32^.

4 $$PE=\left|\frac{{V}_{1}-{V}_{2}}{{V}_{1}}\right|\times 100\%$$

5 $${E}_{a}=\left(\frac{{N}_{1}}{{N}_{2}}\right){RMSE}_{1}+\left(\frac{{N}_{2}}{N}\right){RMSE}_{2}+\left|{RMSE}_{1}-{RMSE}_{2}\right|$$

where ${V}_{1}$ and ${V}_{2}$ represent the target and actual values, respectively. In training and testing datasets, *RMSE*_*1*_ and *RMSE*_*2*_ represent the *RMSE*, while *MAE*_*1*_ and *MAE*_*2*_ represent the *MAE*.

## Results and discussion

### Tuning the electrospun nanofiber membrane diameter

**Figure 3:**
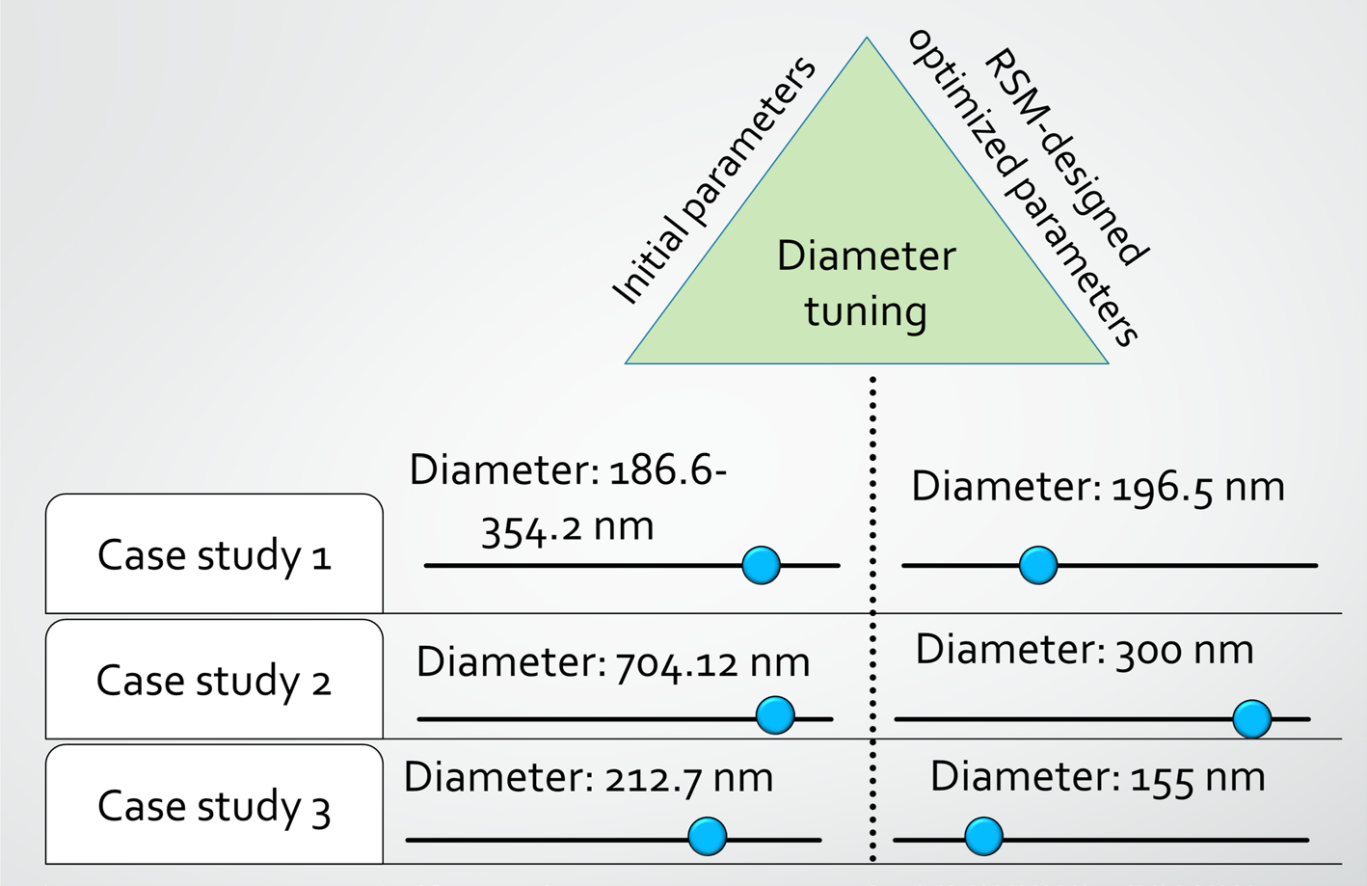
RSM-designed diameter tuning of the electrospun nanofiber membrane.

Computer-based electrospinning replication is crucial. Followingly three case studies were conducted in the current work to understand their simulation behavior along with experimental findings (Fig. 3). Electrospinning was driven by the starting settings in case study 1 generated nanofibers with a mean diameter ranging from 186.6 to 354.2 nm. Following the implementation of the RSM-coupled BBD approach, the optimal conditions were established, and a lower mean diameter of around 196.5 nm was discovered. For case study 2, the minimal diameter was attained with a grand performance average of 704.12 nm; however, following the RSM-linked BBD design, the resultant fiber diameter was decreased to 300 nm, which is a significant improvement. The first selection of electrospinning settings yielded a mean diameter of around 212.7 nm for case study number 3. After that, we were able to generate homogeneous fibers with a mean diameter of 155 nm by optimizing the parameters determined by the RSM-coupled CCD design. Because of the adoption of the RSM optimization approach, the diameter of the electrospun nanofiber membrane was adjusted (smaller is better), which is advantageous for applications involving the treatment of wastewater^47,48^. This was clearly seen in the study that was shown above.

### Principal component analysis for feature selection

**Figure 4:**
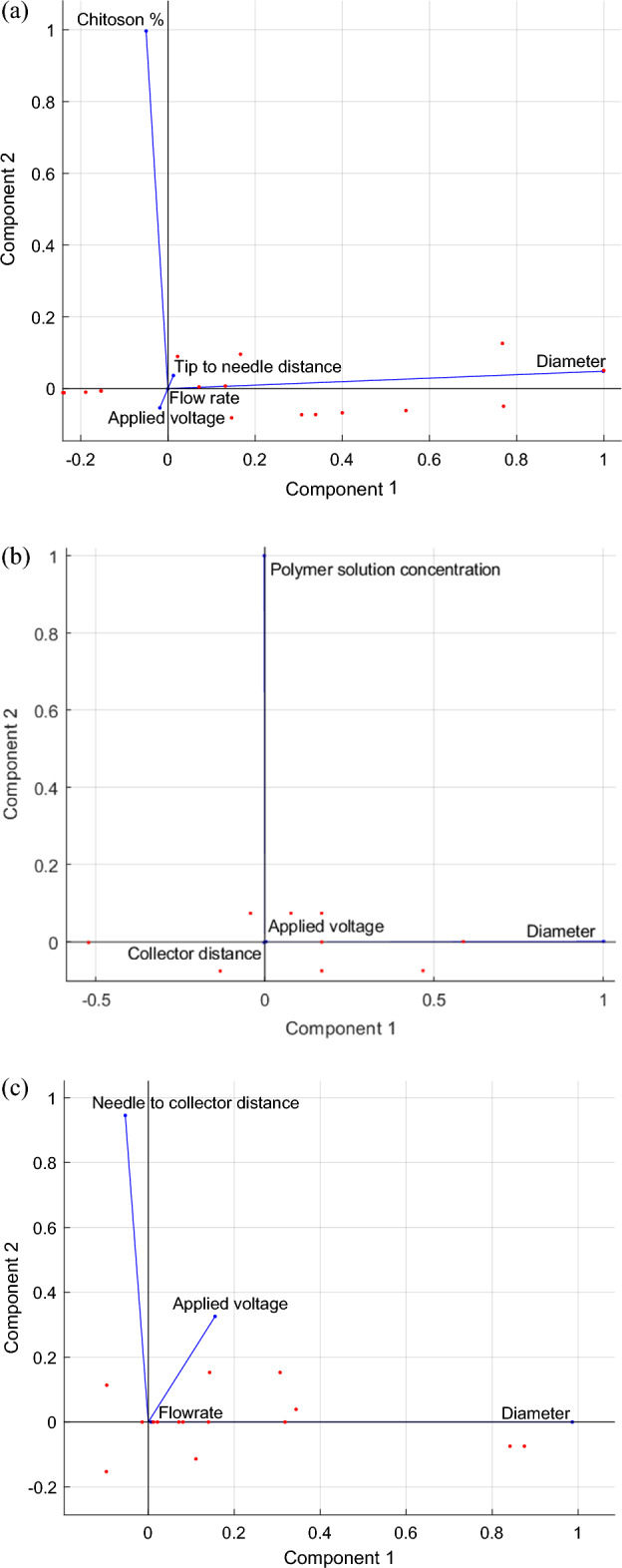
PCA biplot for the electrospinning process of the electrospun nanofiber membrane fabrication (**a**) Case study 1, (**b**) Case study 2, and (**c**) Case study 3.

In the current investigation, principal component analysis (PCA) was used in order to carry out feature selection for the electrospinning process during the manufacturing of electrospun nanofiber membranes. The PCA is a well-known unsupervised dimensionality reduction method that, when applied linearly, generates useful features or variables. The linear combination of the x-variables that have the highest variance is known as the main component^49^. PCA uses the power of eigenvectors and eigenvalues to minimize the quantity of features in a dataset while keeping most of the variance used to quantify the amount of information. This is accomplished via the use of PCA. Therefore, the characteristics or variables that exhibit a larger variation show greater relevance than those that exhibit a lesser variance. In addition to deriving the variance from the PCA, the PCA biplot may be used to show and explain the connection between the variables. The PCA biplot for the electrospinning process of the electrospun nanofiber membrane production for Case studies 1, 2, and 3 is shown in Fig. 4a–c, respectively.

According to the PCA performed on Case study 1, the variances for the input variables, which include the concentration of the chitosan solution, the applied voltage, the tip-to-needle distance, and the flow rate, are, respectively, 46.1538, 2.8846, 2.8846, and 0.0288. As a result, the first three input variables, which are the concentration of the chitosan solution, the applied voltage, and the tip-to-needle distance, are significant since they account for 99.94% of the variance. Figure 4a for Case study 1 likewise reveals comparable findings; however, except for flow rate, the first three input variables include more information. The PCA for Case study 2 reveals that the polymer solution concentration, the applied voltage, and the collector distance have relative variances of 312.5, 12.5 and 8, respectively. As a result, the first two input variables, the polymer solution concentration and the voltage applied are significant since they account for 97.60% of the variance. Due to the fact that they both have the largest degree of variance, the findings of Case study 2 demonstrate that the concentration of the polymer solution and the applied voltage provide more information than the other variables (Fig. 4b). Finally, PCA found that the variances of the input variables applied voltage, flow rate, and needle-collector distance were 28.3575, 0.1175, and 0 for Case study 3. These findings suggest that the applied voltage and flow rate account for up to 100% of the variation, whereas the needle-collector distance provides no useful information. Figure 4c for Case study 3 demonstrates that input factors, such as applied voltage and flow rate, offer more insight than the needle-collector distance.

Based on the results of the three case studies with varying experimental designs, it is possible to conclude the critical input variables of the electrospinning process necessary for producing an electrospun nanofiber membrane.

### Modelling assessment

In this work, the experimental process data for the electrospinning technique that yields nanofibers was utilized to build LSSVR, PCR, PLSR, LW-PLSR, LW-KPLSR, and Fuzzy models. These models were used to predict the results of the study. These models, which are referred to as artificial intelligence (AI) or machine learning (ML) models, may be utilized for process optimization to acquire the desired outcomes by monitoring the process data. In this part, the modelling evaluation was carried out to understand the capabilities of various AI models to select and introduce the best AI model for the optimization of the electrospinning process to support laboratory and industrial trials.

In order to assess the efficacy of these AI models, three distinct case studies for the electrospinning process of producing nanofibers were used. The outcomes of these case studies were reported in terms of *RMSE*, *MAE*, *R*_*2*_*,* and *Ea*, and they were organized and shown in Tables 2, 3, 4. As was discussed previously in "Analysis of prediction behaviour" section, these tables' *RMSE*_*1*_, *MAE*_*1*_, *RMSE*_*2*_, and *MAE*_*2*_ columns represent the mean absolute error (MAE) and root-mean-square error (RMSE) for training and testing data, respectively. In addition to the RMSE and MAE, the *R*^*2*^ value is denoted as the *R*_*1*_^*2*^ and *R*_*2*_^*2*^, respectively, for training data and testing data. The findings for Case study 1 are presented in Table 2, and it is clear that the LW-KPLSR provides the best results when compared to the other models thanks to its low Ea value of 25.4921 and its high *R*_*1*_^*2*^ and *R*_*2*_^*2*^ values of 0.8534 and 0.7509. The overall performance of LW-KPLSR is greater, whereas LW-PLSR delivers better *RMSE*_*1*_, *MAE*_*1*_, and *R*_*1*_^*2*^.

**Table 2:** Predictive modelling results for Poly (vinyl alcohol)/chitosan crosslinked electrospun nanofiber diameter (Case study 1).

Models	LW-KPLSR	LSVVR	PE (%)	Fuzzy method	PE (%)	PLSR	PE (%)	PCR	PE (%)	LW-PLSR	PE (%)
Training data											
* RMSE*_*1*_	16.6615	25.5405	− 53	16.1874	3	47.0458	− 182	48.4920	− 191	3.6995	78
* MAE*_*1*_	12.8781	20.3188	− 58	12.9889	− 1	38.0012	− 195	39.4215	− 206	2.3421	82
* R*_*1*_^*2*^	0.8534	0.5945	30	0.9198732	− 8	− 4.3966	615	− 7.6465	996	0.9948	− 16.57
Testing data											
* RMSE*_*2*_	23.2845	22.7570	2	50.5757	− 117	33.4241	− 44	34.4090	− 48	26.7814	− 15
* MAE*_*2*_	21.0767	18.6228	12	42.2333	− 100	27.7003	− 31	28.8202	− 37	22.4287	− 6
* R*_*2*_^*2*^	0.7509	0.7354	2	− 0.8718	216	− 0.5223	170	− 0.8531	214	0.4190	44
* E*_*a*_	25.4921	27.3299	− 7	62.0384	− 143	56.1270	− 120	57.8808	− 127	34.4754	− 35

**Table 3:** Predictive modelling results for chitosan-based electrospun nanofiber diameter (Case study 2).

Models	LW-KPLSR	LSVVR	PE (%)	Fuzzy method	PE (%)	PLSR	PE (%)	PCR	PE (%)	LW-PLSR	PE (%)
Training data											
* RMSE*_*1*_	5.6667	5.2597	7	64.9678	− 1046	173.1629	− 2956	173.3743	− 2960	12.4353	− 119
* MAE*_*1*_	3.1167	4.7538	− 53	51.3636	− 1548	155.3850	− 4886	155.8589	− 4901	9.1219	− 193
* R*_*1*_^*2*^	0.9989	0.9990	0	0.8877	11	− 150.3780	15,154	− 239.8229	24,108	0.9943	0.47
Testing data											
* RMSE*_*2*_	42.5396	79.5503	− 87	72.1757	− 70	55.5535	− 31	242.4839	− 470	40.7959	4
* MAE*_*2*_	33.6509	51.3537	− 53	64.6667	− 92	234.6380	− 597	234.2000	− 596	25.4019	25
* R*_*2*_^*2*^	0.8115	0.6616	18	0.6224	23	− 0.0702	109	− 0.0715	109	0.7048	13
* E*_*a*_	55.5535	105.7705	− 90	74.7197	− 35	267.4248	− 381	266.8756	− 380	50.8055	9

**Table 4:** Predictive modelling results for chitosan-collagen electrospun nanofiber diameter (Case study 3).

Models	LW-KPLSR	LSVVR	PE (%)	Fuzzy method	PE (%)	PLSR	PE (%)	PCR	PE (%)	LW-PLSR	PE (%)
Training data											
* RMSE*_*1*_	8.8612	1.0489	88	16.3039	− 84	11.1017	− 25	13.7622	− 55	3.0948	65
* MAE*_*1*_	6.3450	0.7241	89	12.2615	− 93	8.5467	− 35	11.1910	− 76	1.9152	70
* R*_*1*_^*2*^	0.7015	0.9970	− 42	− 0.7000	200	0.4915	30	− 0.0748	111	0.9707	− 38.37
Testing data											
* RMSE*_*2*_	7.1471	26.4967	− 271	17.6887	− 147	5.5468	22	13.4870	− 89	6.3825	11
* MAE*_*2*_	5.8213	21.1671	− 264	13.7143	− 136	5.3270	8	12.7765	− 119	5.3595	8
* R*_*2*_^*2*^	0.7805	0.3306	58	− 0.7406	195	0.8145	− 4	0.3982	49	0.7747	1
* E*_*a*_	9.9753	35.4035	− 255	18.1734	− 82	14.7124	− 47	13.9411	− 40	7.5332	24

Table 2 further demonstrates the effectiveness of both LW-KPLSR and LW-PLSR, with *RMSE*_*1*_ and *MAE*_*1*_ values around 1 and *R*_*1*_^*2*^ values greater than 0.999. This is owing to the fact that both models make use of the same integrated model, specifically the locally weighted (LW) algorithm^50^, which employs a weighted Euclidean distance-based strategy to choose the proper historical data that leads to excellent prediction. However, compared to LW-KPLSR, its *RMSE*_*2*_, *MAE*_*2,*_ and *R*_*2*_^*2*^ scores are 15% to 44% lower for the testing data because the LW-PLSR does not have a kernel function. Equation (6)^51^ illustrates the use of a Rational quadratic (RQ) kernel in the LW-KPLSR model for the first case study.

6 $$k(\text{x,}{x}^{^{\prime}})=1-\frac{{\Vert x-{x}^{^{\prime}}\Vert }^{2}}{{\Vert x-{x}^{^{\prime}}\Vert }^{2}+b}$$

where ${\left|x-{x}^{^{\prime}}\right|}^{2}$ is the Euclidean distance of the binary vectors *x* and *x′*, and b is the kernel parameter. When the distance between the vectors *x* and *x′* becomes higher, then the value of the RQ function will be consistently increasing. The inclusion of the RQ kernel function in the LW-KPLSR model brings about a general reduction in the complexity of the model and helps to prevent the occurrence of small sample overfitting in the model^51^. According to Table 2, alternative models, such as LSSVR, PCR, PLSR, and fuzzy approaches, all produced unsatisfactory results due to the fact that their *RMSE*_*1*_ and *MAE*_*1*_ values are between 8 and 206% worse than the results produced by the LW-KPLSR model. In the meanwhile, other models, such as the LW-PLSR, have shown poor results and have values that are 15% to 100% higher for *RMSE*_*2*_, *MAE*_*2*_, and *E*_*a*_ values and that are 44% to 216% lower for *R*_*2*_^*2*^ in comparison to the LW-KPLSR.

On the other hand, the findings of Case study 2 are shown in Table 3, and once again, LW-KPLSR performed better than the other models. Table 3 demonstrates, in a manner similar to that of Case study 1, that the outcomes of the training data for LW-KPLSR and LW-PLSR are superior to those obtained by using other techniques. Because the LW model is present, the *RMSE*_*1*_* and MAE*_*1*_ values for these individuals are less than 1, and the *R*_*1*_^*2*^ values for these individuals are more than 0.999. Again, when compared to other models, the LW-KPLSR achieves superior overall outcomes with its Ea value, which has values that are 13% to 597% lower than those of the other models. In the second case study, LW-KPLSR made use of the Polynomial Kernel function, which can be found outlined in Eq. (7)^52^.

7 $$k(x\text{,}{ x}^{^{\prime}})={\left({x}^{T}{x}^{^{\prime}}+1\right)}^{b}$$

In addition, Yeo et al.^33^, Yeo et al.^53^, and Yeo et al.^52^ have shown that the LW-KPLSR model with the Polynomial Kernel has an excellent predictive performance. In Case study 2, the Polynomial Kernel was used to map the nonlinear data produced by the electrospinning method into a space with a greater dimension in order to generate more accurate predicted findings.

In addition to LW-KPLSR and LW-PLSR, Table 3 also includes the *R*_*1*_^*2*^ value for the fuzzy approach, which is 0.8877. This value is also satisfactory. The fuzzy technique may provide an approximation of the fuzzy connections that exist between the variables that are independent and those that are dependent^54^. Despite this, the values of *RMSE*_*1*_ and *MAE*_*1*_ for the fuzzy technique are 1046% and 1548% higher, respectively, than those for LW-KPLSR. In addition, the values of *E*_*a*_, *RMSE*_*2*_, and *MAE*_*2*_ that are produced by the fuzzy technique are between 35 and 92% higher than those created by LW-KPLSR. This is because the fuzzy logic system uses a fuzzy rule base, which only includes fuzzy IF-THEN rules that are produced from the training data^55^. As a result, the fuzzy technique loses its capacity for appropriate precision if the data being tested are distinct from the data being used for training.

In Case study 2, in addition to the LW-KPLSR, the LW-PLSR, and the fuzzy approach, the LSSVR offered excellent results for the training data. Its *R*_*1*_^*2*^ value is 0.9990, and its *RMSE*_*1*_ and *MAE*_*1*_ values are pretty modest. These numbers indicate that the LSSVR performed well. The LSSVR, much like the LW-KPLSR, makes use of a kernel function. This kernel function, known as the radial basis function kernel, provides an approximate feature map in a limited number of dimensions for the nonlinear data^56^. In spite of this, looking at Table 3, we can see that the *E*_*a*_, *RMSE*_*2*_, and *MAE*_*2*_ values of the LSSVR are 53–90% higher than those of the LW–KPLSR. This is possible because of the assistance provided by the LW method and the polynomial kernel function included in the LW-KPLSR. According to Table 3, and similarly to the findings of Case study 1, it is clear that PLSR and PCR did not provide satisfactory outcomes because they are linear models that cannot deal with the nonlinear data produced by the electrospinning process^44^.

On the other hand, the findings for Case study 3 are shown in Table 4. These results were obtained using LW-KPLSR, LSSVR, the Fuzzy technique, PLSR, PCR, and LW-PLSR. Case study 3 has a total of 20 datasets, and it is clear from looking at Table 4 that the findings for the testing data, particularly the *R*_*2*_^*2*^ values for LSSVR and PLSR, are much improved (non-negative values). The findings of Case study 3 for the training data from LW-PLSR and LSSVR seem to be positive, the same as the results of Case study 2, since their *R*_*1*_^*2*^ values are higher than 0.95. Meanwhile, these models are not as good as LW-KPLSR because their *E*_*a*_, *RMSE*_*2*_, *MAE*_*2*_ and *R*_*2*_^*2*^ values are lower than LW-KPLSR by 89% to 255%. Surprisingly, the results of *RMSE*_*2*_ and *MAE*_*2*_ for the testing data for PLSR and LW-PLSR are almost equal. According to these findings, the LW method used in LW-PLSR does not significantly improve the prediction in Case study 3. However, LW-KPLSR has both an LW algorithm and a kernel function, a Multi Quadric (MQ) kernel (as indicated in Eq. 8), making it a unique system. This MQ kernel translates the original nonlinear data into higher dimensional space, which ultimately results in improved predictive performance. Similar to Case studies 1 and 2, LW-KPLSR is exceptional in comparison to other models because it achieves superior overall outcomes, as shown by its lower Ea values and the fact that both *R*_*1*_^*2*^ and *R*_*2*_^*2*^ are higher than the benchmarked value of 0.6 for R^2^.

8 $$k({x,x}^{^{\prime}})=\sqrt{{\Vert x-{x}^{^{\prime}}\Vert }^{2}+{b}^{2}}$$

### Predictivity assessment

**Figure 5:**
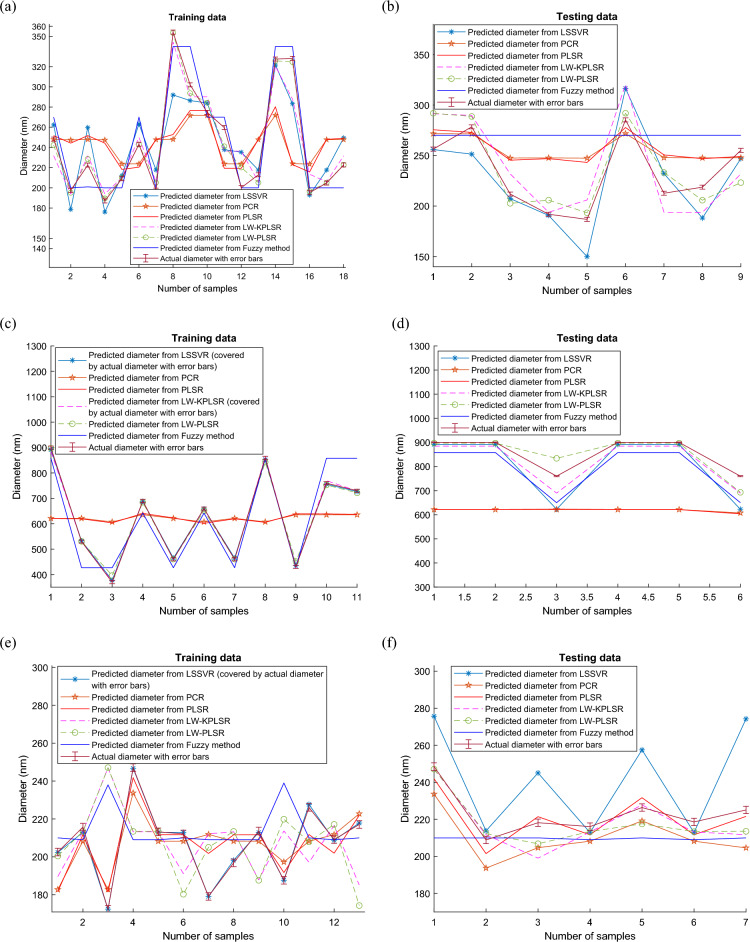
Comparison of predictive results from LSSVR, PCR, PLSR, LW-PLSR, LW-KPLSR and fuzzy models (**a**) Case study 1 training data, (**b**) Case study 1 testing data, (**c**) Case study 2 training data, (**d**) Case study 2 testing data, (**e**) Case study 3 training data, and (**f**) Case study 3 testing data.

This part displays the graphs that compare the predicted outcomes of LW-KPLSR, LW-PLSR, PCR, PLSR, Fuzzy technique, and LSSVR for Case studies 1 to 3. The prediction outputs from the training data of case studies 1, 2, and 3 are compared in Fig. 5a,c,e, respectively. In the meanwhile, the comparison of the predicted outputs for the testing data of case studies 1, 2, and 3 is shown in Fig. 5b,d,f, respectively. It is clear to observe that the anticipated outputs from LW-PLSR and LW-KPLSR shown in Fig. 5a,c,e are highly similar to the actual data, which is the diameter of the nanofiber expressed in nanometers. In addition to this, all of the predicted data from the LW-PLSR and the LW-KPLSR can be shown to fall inside the error bars for the actual data in Fig. 5a,c,e. The locally weighted model, which uses historical data close to the query sample of the intended output variables^57^, was vital in producing these findings. However, with the assistance of the kernel function, the predicted data from LW-KPLSR in Fig. 5b,d,f are closer to the actual data when compared to the predicted data from other models, including LW-PLSR. In addition, the results of PLSR, PCR, and fuzzy approach predictions, as shown in Fig. 5b,d, are pretty different from the actual data and associated error bars. Because of this, it is impossible to utilize them to predict the nanofiber diameter using them accurately.

Compared to the other models, the LW-KPLSR provided the predicted outputs that are either closer to or fall within the error bars of the actual data, as seen in Fig. 5a through 5f. These results pertain to the overall findings. The LW-KPLSR can provide satisfactory results despite the limited experimental data that are currently available for the electrospinning technique that is used to fabricate nanofibers.

The R^2^ values of LW-KPLSR, displayed in Tables 2, 3, 4, are greater than 0.6, a benchmark for good external predictability^58^. In addition, the outcomes of the LW-KPLSR model have the potential to be enhanced further by the collection of more datasets. As a result, one may conclude that the LW-KPLSR model is an appropriate choice to optimize the process data for the electrospinning method.

### Accuracy of the models

**Figure 6:**
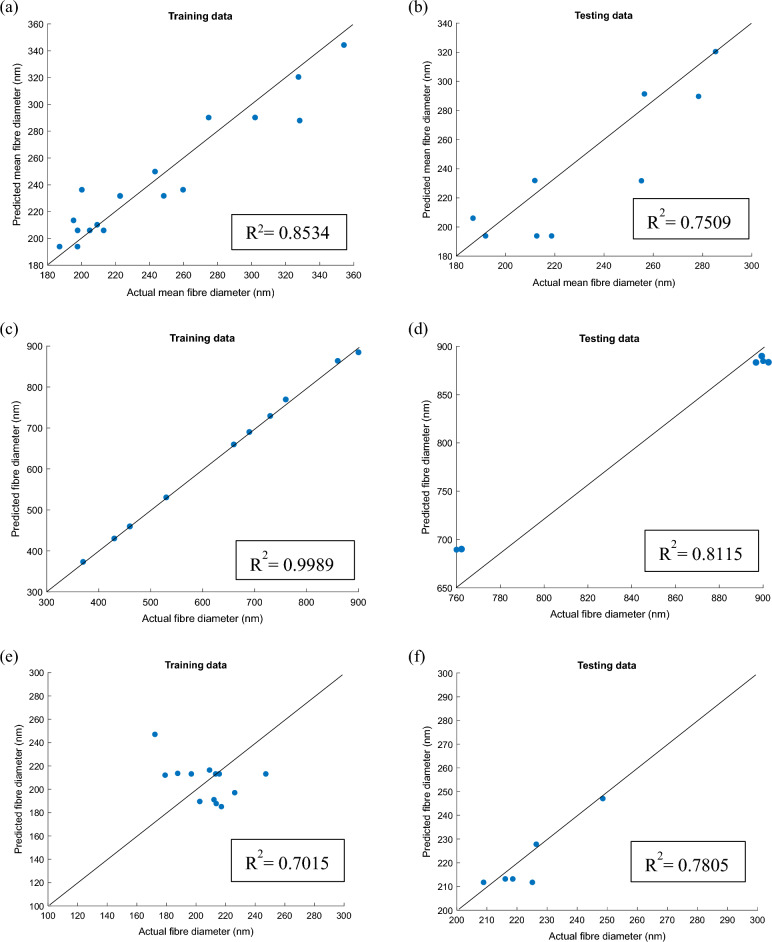
Correlation between the actual and predicted values from the LW-KPLSR model (**a**) Case study 1 using training data, (**b**) Case study 1 using testing data, (**c**) Case study 2 using training data, (**d**) Case study 2 using testing data, (**e**) Case study 3 using training data, (**f**) Case study 3 using testing data.

In this subsection, Fig. 6a–f illustrate the correlation between the actual and predicted values for the training and testing data derived from the LW-KPLSR model. It is important to take note of the fact that the actual and predicted data generated by LW-KPLSR and shown in Fig. 6a,c,e for the training data are quite similar to one another, as seen by their R^2^ values, which are all greater than 0.7. Because the training data are used to create and assess the LW-KPLSR, as shown in Fig. 2, the findings are often less significant than the testing data^59^. This is because the training data are used. In the meanwhile, the testing data that was not included in the construction of the LW-KPLSR model may be found depicted in Fig. 6b,d,f, respectively. It is clear from these figures that some of the projected outputs are quite a distance from the y-x line; despite this, the R2 values for the testing data are still greater than the R^2^ values that were used as a benchmark. In conclusion, the LW-KPLSR model is appropriate for predicting the nanofiber's diameter.

## Conclusions

In conclusion, the present research produced a unique combination method, the RSM-integrated LW-KPLSR model, for predicting the diameter of the electrospun nanofiber membranes. This was accomplished by applying the electrospinning process data from three case studies. In case study 1, the variables that are considered to be input are the applied voltage, flow rate, chitosan solution concentration, and tip-to-needle distance. In case study 2, input variables are the collector distance, polymer solution concentration, and applied voltage, and for case study 3, the applied voltage, flow rate, and needle-to-collector distance. It is essential to ascertain the optimal electrospinning process parameters to attain the smallest nanofiber diameter for the membrane. As a result, the implementation of the RSM design was appropriate. The RSM design and predictive modelling techniques such as LW-KPLSR play an important role in producing smaller-sized diameter-based electrospun nanofiber membranes. The LW-KPLSR model fared much better overall at predicting the fibre's diameter than the Fuzzy approach, the PCR model, the LW-PLSR model, the PLSR model, and the LSSVR model. This is shown by the LW-KPLSR model's lower *E*_*a*_ values. Additionally, the R^2^ values that it generates in each case study are high, with some reaching as high as 0.9989. In light of these discoveries, it has come to light that the LW-KPLSR model may be put to work in electrospinning nanofiber membranes in the capacity of a diameter prediction instrument. Further study may reveal that including a locally weighted algorithm in the LW-KPLSR model improves the model's ability to make accurate predictions. The findings of this research shed light on the significance of achieving long-term sustainability and cost reductions via the integration of RSM and AI to swiftly optimize the electrospinning process and generate the intended membrane diameter shape.

## Supplementary Information

Supplementary Tables.

## Data Availability

The datasets generated during the current study are available from the corresponding author on reasonable request (Prof. Yingjie Cai, Y. Cai).

## Code availability

No code was attempted or used during the current manuscript.
