# Supplementary material for: Electrospun nanofiber membrane diameter prediction using a combined response surface methodology and machine learning approach
Source: Sci Rep. 2023 Jun 15;13:9679. doi: 10.1038/s41598-023-36431-7 (PMC10272235; doi:10.1038/s41598-023-36431-7)
Supplement: Supplementary file 1 — Supplementary Tables. [file 41598_2023_36431_MOESM1_ESM.docx]

**Supplementary file**

**Electrospun nanofiber membrane diameter prediction using a combined response surface methodology and machine learning approach**

Md. Nahid Pervez^a,b^, Wan Sieng Yeo^c^, Mst. Monira Rahman Mishu^d^, Md. Eman Talukder^a^, Hridoy Roy^e^, Md. Shahinoor Islam^e^, Yaping Zhao^f^, Yingjie Cai^a*^, George K Stylios^g*^, Vincenzo Naddeo^b*^

^a^ Hubei Provincial Engineering Laboratory for Clean Production and High Value Utilization of Bio-based Textile Materials, Wuhan Textile University, Wuhan 430200, China

^b^ Sanitary Environmental Engineering Division (SEED), Department of Civil Engineering, University of Salerno, Fisciano 84084, Italy

^c^ Department of Chemical and Energy Engineering, Faculty of Engineering and Science, Curtin University Malaysia, CDT 250, 98009 Miri, Sarawak, Malaysia

^d^ Faculty of Nutrition and Food Science, Patuakhali Science and Technology University, Patuakhali 8602, Bangladesh

^e^ Department of Chemical Engineering, Bangladesh University of Engineering and Technology, Dhaka 1000, Bangladesh

^f^ Shanghai Engineering Research Center of Biotransformation of Organic Solid Waste, School of Ecological and Environmental Sciences, East China Normal University, and Institute of Eco-Chongming, Shanghai, 200241, China

^g^ Research Institute for Flexible Materials, School of Textiles and Design, Heriot-Watt University, Galashiels, TD1 3HF, UK

**^*^ Corresponding authors:**

[yingjiecai@wtu.edu.cn](mailto:yingjiecai@wtu.edu.cn) (Y. Cai) ; [g.stylios@hw.ac.uk](mailto:g.stylios@hw.ac.uk) (G.K. Stylios); [vnaddeo@unisa.it](mailto:vnaddeo@unisa.it) (V. Naddeo)

**Lists of the supplementary materials**

**Table S1.** Box-Behnken Design and experimental response (Case study 1)

**Table S2.** Box-Behnken Design and experimental response (Case study 2)

**Table S3.** Central composite design and experimental response (Case study 3)

**Table S4.** Dataset for model development (Case study 1)

**Table S5.** Dataset for model development (Case study 2)

**Table S6.** Dataset for model development (Case study 3)

**Table S1.** Box-Behnken Design and experimental response (Case study 1)

| No. | Level of factors | | | | Response |
| --- | --- | --- | --- | --- | --- |
|  | Applied voltage (kV) | Flow rate (mL/h) | Chitosan (%) | Tip to needle distance (cm) | Mean fiber diameter (nm) |
| 1 | 10 | 0.25 | 30 | 12.5 | 248.3 |
| 2 | 15 | 0.25 | 30 | 12.5 | 197.6 |
| 3 | 10 | 0.75 | 30 | 12.5 | 222.6 |
| 4 | 15 | 0.75 | 30 | 12.5 | 187.1 |
| 5 | 12.5 | 0.5 | 20 | 10 | 256.4 |
| 6 | 12.5 | 0.5 | 40 | 10 | 209.2 |
| 7 | 12.5 | 0.5 | 20 | 15 | 285.3 |
| 8 | 12.5 | 0.5 | 40 | 15 | 243.2 |
| 9 | 12.5 | 0.5 | 30 | 12.5 | 197.7 |
| 10 | 10 | 0.5 | 30 | 10 | 212.7 |
| 11 | 15 | 0.5 | 30 | 10 | 186.8 |
| 12 | 10 | 0.5 | 30 | 15 | 354.2 |
| 13 | 15 | 0.5 | 30 | 15 | 211.9 |
| 14 | 12.5 | 0.25 | 20 | 12.5 | 302 |
| 15 | 12.5 | 0.75 | 20 | 12.5 | 274.7 |
| 16 | 12.5 | 0.25 | 40 | 12.5 | 259.7 |
| 17 | 12.5 | 0.75 | 40 | 12.5 | 200.2 |
| 18 | 12.5 | 0.5 | 30 | 12.5 | 213 |
| 19 | 10 | 0.5 | 20 | 12.5 | 327.5 |
| 20 | 15 | 0.5 | 20 | 12.5 | 278.4 |
| 21 | 10 | 0.5 | 40 | 12.5 | 328.2 |
| 22 | 15 | 0.5 | 40 | 12.5 | 195.3 |
| 23 | 12.5 | 0.25 | 30 | 10 | 191.9 |
| 24 | 12.5 | 0.75 | 30 | 10 | 218.7 |
| 25 | 12.5 | 0.25 | 30 | 15 | 222.7 |
| 26 | 12.5 | 0.75 | 30 | 15 | 255.2 |
| 27 | 12.5 | 0.5 | 30 | 12.5 | 204.8 |

**Table S2.** Box-Behnken Design and experimental response (Case study 2)

| No. | Level of factors | | | Response |
| --- | --- | --- | --- | --- |
|  | Collector distance (cm) | Polymer solution concentration (wt%) | Applied voltage (kV) | Mean fiber diameter (nm) |
| 1 | 9 | 50 | 10 | 900 |
| 2 | 9 | 50 | 10 | 900 |
| 3 | 9 | 50 | 10 | 900 |
| 4 | 13 | 50 | 5 | 530 |
| 5 | 13 | 25 | 10 | 370 |
| 6 | 5 | 50 | 15 | 760 |
| 7 | 9 | 75 | 15 | 690 |
| 8 | 13 | 50 | 15 | 460 |
| 9 | 9 | 25 | 5 | 660 |
| 10 | 9 | 50 | 10 | 900 |
| 11 | 5 | 50 | 5 | 460 |
| 12 | 9 | 50 | 10 | 900 |
| 13 | 9 | 25 | 15 | 860 |
| 14 | 5 | 25 | 10 | 760 |
| 15 | 5 | 75 | 10 | 430 |
| 16 | 13 | 75 | 10 | 760 |
| 17 | 9 | 75 | 5 | 730 |

**Table S3.** Central composite design and experimental response (Case study 3)

| No. | Level of factors | | | Response |
| --- | --- | --- | --- | --- |
|  | Applied voltage kV | Flow rate  mL/h | Distance (cm) | Mean fiber diameter (nm) |
| Training data for model development | | | | |
| 1 | 10 | 1.5 | 25 | 202.5 |
| 2 | 15 | 1 | 20 | 215.7 |
| 3 | 10 | 0.5 | 25 | 172.2 |
| 4 | 20 | 1.5 | 15 | 248.5 |
| 5 | 20 | 0.5 | 15 | 247.1 |
| 6 | 15 | 1 | 20 | 213.2 |
| 7 | 15 | 1 | 20 | 212.1 |
| 8 | 10 | 0.5 | 15 | 179.1 |
| 9 | 15 | 0.5 | 20 | 196.8 |
| 10 | 15 | 1 | 20 | 213.6 |
| 11 | 10 | 1 | 20 | 187.6 |
| 12 | 15 | 1.5 | 20 | 226.1 |
| 13 | 20 | 0.5 | 25 | 218.2 |
| 14 | 15 | 1 | 15 | 217.1 |
| 15 | 15 | 1 | 25 | 208.9 |
| 16 | 10 | 1.5 | 15 | 209.1 |
| 17 | 15 | 1 | 20 | 216.1 |
| 18 | 20 | 1 | 20 | 226.4 |
| 19 | 15 | 1 | 20 | 218.6 |
| 20 | 20 | 1.5 | 25 | 225.1 |

**Table S4.** Dataset for model development (Case study 1)

|  | Observed variables | | | | Targeted variables |
| --- | --- | --- | --- | --- | --- |
| No. | Input 1 | Input 2 | Input 3 | Input 4 | Output 1 |
|  | Applied voltage kV | Flow rate mL/h | Chitosan % | Tip to needle distance (cm) | Mean fiber diameter (nm) |
| Training data for model development | | | | | |
| 1 | 10 | 0.25 | 30 | 12.5 | 248.3 |
| 2 | 15 | 0.25 | 30 | 12.5 | 197.6 |
| 3 | 10 | 0.75 | 30 | 12.5 | 222.6 |
| 4 | 15 | 0.75 | 30 | 12.5 | 187.1 |
| 6 | 12.5 | 0.5 | 40 | 10 | 209.2 |
| 8 | 12.5 | 0.5 | 40 | 15 | 243.2 |
| 9 | 12.5 | 0.5 | 30 | 12.5 | 197.7 |
| 12 | 10 | 0.5 | 30 | 15 | 354.2 |
| 14 | 12.5 | 0.25 | 20 | 12.5 | 302 |
| 15 | 12.5 | 0.75 | 20 | 12.5 | 274.7 |
| 16 | 12.5 | 0.25 | 40 | 12.5 | 259.7 |
| 17 | 12.5 | 0.75 | 40 | 12.5 | 200.2 |
| 18 | 12.5 | 0.5 | 30 | 12.5 | 213 |
| 19 | 10 | 0.5 | 20 | 12.5 | 327.5 |
| 21 | 10 | 0.5 | 40 | 12.5 | 328.2 |
| 22 | 15 | 0.5 | 40 | 12.5 | 195.3 |
| 27 | 12.5 | 0.5 | 30 | 12.5 | 204.8 |
| 25 | 12.5 | 0.25 | 30 | 15 | 222.7 |
| Testing data for model validation | | | | | |
| 5 | 12.5 | 0.5 | 20 | 10 | 256.4 |
| 20 | 15 | 0.5 | 20 | 12.5 | 278.4 |
| 13 | 15 | 0.5 | 30 | 15 | 211.9 |
| 23 | 12.5 | 0.25 | 30 | 10 | 191.9 |
| 11 | 15 | 0.5 | 30 | 10 | 186.8 |
| 7 | 12.5 | 0.5 | 20 | 15 | 285.3 |
| 10 | 10 | 0.5 | 30 | 10 | 212.7 |
| 24 | 12.5 | 0.75 | 30 | 10 | 218.7 |
| 26 | 12.5 | 0.75 | 30 | 15 | 255.2 |

**Table S5.** Dataset for model development (Case study 2)

|  | Observed variables | | | Targeted variables |
| --- | --- | --- | --- | --- |
| No. | Input 1 | Input 2 | Input 3 | Output 1 |
|  | Collector distance (cm) | Polymer solution concentration (wt%) | Applied voltage (kV) | Nanofiber diameter (nm) |
| Training data for model development | | | | |
| 2 | 9 | 50 | 10 | 900 |
| 4 | 13 | 50 | 5 | 530 |
| 5 | 13 | 25 | 10 | 370 |
| 7 | 9 | 75 | 15 | 690 |
| 8 | 13 | 50 | 15 | 460 |
| 9 | 9 | 25 | 5 | 660 |
| 11 | 5 | 50 | 5 | 460 |
| 13 | 9 | 25 | 15 | 860 |
| 15 | 5 | 75 | 10 | 430 |
| 16 | 13 | 75 | 10 | 760 |
| 17 | 9 | 75 | 5 | 730 |
| Testing data for model validation | | | | |
| 1 | 9 | 50 | 10 | 900 |
| 3 | 9 | 50 | 10 | 900 |
| 6 | 5 | 50 | 15 | 760 |
| 10 | 9 | 50 | 10 | 900 |
| 12 | 9 | 50 | 10 | 900 |
| 14 | 5 | 25 | 10 | 760 |

**Table S6.** Dataset for model development (Case study 3)

|  | Observed variables | | | Targeted variables |
| --- | --- | --- | --- | --- |
| No. | Input 1 | Input 2 | Input 3 | Output 1 |
|  | Applied voltage kV | Flow rate mL/h | Distance (cm) | Mean fiber diameter (nm) |
| Training data for model development | | | | |
| 1 | 10 | 1.5 | 25 | 202.5 |
| 2 | 15 | 1 | 20 | 215.7 |
| 5 | 20 | 0.5 | 15 | 247.1 |
| 6 | 15 | 1 | 20 | 213.2 |
| 4 | 20 | 1.5 | 15 | 248.5 |
| 7 | 15 | 1 | 20 | 212.1 |
| 8 | 10 | 0.5 | 15 | 179.1 |
| 9 | 15 | 0.5 | 20 | 196.8 |
| 10 | 15 | 1 | 20 | 213.6 |
| 11 | 10 | 1 | 20 | 187.6 |
| 13 | 20 | 0.5 | 25 | 218.2 |
| 15 | 15 | 1 | 25 | 208.9 |
| 20 | 20 | 1.5 | 25 | 225.1 |
| Testing data for model validation | | | | |
| 3 | 10 | 0.5 | 25 | 172.2 |
| 12 | 15 | 1.5 | 20 | 226.1 |
| 14 | 15 | 1 | 15 | 217.1 |
| 16 | 10 | 1.5 | 15 | 209.1 |
| 17 | 15 | 1 | 20 | 216.1 |
| 18 | 20 | 1 | 20 | 226.4 |
| 19 | 15 | 1 | 20 | 218.6 |
